# Development and Optimization of Nanoemulsion from Ethanolic Extract of *Centella asiatica* (NanoSECA) Using D-Optimal Mixture Design to Improve Blood-Brain Barrier Permeability

**DOI:** 10.1155/2022/3483511

**Published:** 2022-03-07

**Authors:** Nor Atiqah Jusril, Syahrul Imran Abu Bakar, Khalilah Abdul Khalil, Wan Mazlina Md Saad, Ng Kwok Wen, Mohd Ilham Adenan

**Affiliations:** ^1^Faculty of Applied Sciences, Universiti Teknologi MARA, Shah Alam 40450, Selangor Darul Ehsan, Malaysia; ^2^Atta-ur-Rahman Institute for Natural Product Discovery (AuRIns), Level 9, FF3, Puncak Alam Campus, Universiti Teknologi MARA, Puncak Alam 42300, Selangor Darul Ehsan, Malaysia; ^3^Centre of Medical Laboratory Technology, Faculty of Health Sciences, Universiti Teknologi MARA, Puncak Alam 42300, Selangor Darul Ehsan, Malaysia; ^4^Faculty of Pharmacy, Quest International University, Jalan Raja Permaisuri Bainun, 30250 Ipoh, Perak, Malaysia; ^5^Universiti Teknologi MARA Pahang Branch, Bandar Pusat Jengka 26400, Pahang Darul Makmur, Malaysia

## Abstract

The evidence on the neuroprotective impact of *Centella asiatica* (*C. asiatica*) has been greatly documented in recent years. However, a major obstacle that remains to be overcome is the capacity of the active molecules in *C. asiatica* to cross the blood-brain barrier (BBB). In this study, we explored the possibilities of using a D-optimal mixture design to fabricate nanoemulsion of *C. asiatica* (NanoSECA) for better brain bioavailability. The parameters for optimization were the percentage of water (10–80% w/v) and virgin coconut oil (VCO) (10–80% w/v). Nanoemulsions were formulated using a high-pressure homogenization approach and were characterized for their physicochemical properties. The optimal VCO-based nanoemulsion (VBN: F2) conditions were found at 80% (w/v) of water and 10% (w/v) of VCO. Subsequently, viability tests were conducted on neuroblastoma (SH-SY5Y) and macrophage (RAW 264.7) cell lines. NanoSECA was distinguished for its antioxidant, acetylcholinesterase (AChE), anti-inflammatory, and parallel artificial membrane permeability assay (PAMPA) activities *in vitro*. The NanoSECA has a particle size of 127.833 ± 8.280 nm, zeta potential (ZP) of −24.9 ± 0.011 mV, polydispersity index (PDI) of 0.493 ± 4.681, percentage prediction error (PPE) of −12.02%, and pH of 6.0 ± 0.006 and is also stable under different storage conditions. Cell viability was improved in a dose-dependent manner on SH-SY5Y and RAW 264.7 cell lines. In addition, NanoSECA significantly reduced the AChE activity, suppressing the level of proinflammatory mediators and oxidative stress. Moreover, NanoSECA showed high BBB permeation with a high value of experimental permeability to cross the BBB. Thus, NanoSECA could efficiently potentiate the central nervous system (CNS) therapeutic activities through enhanced penetration of BBB. These nano-delivery systems are crucial to unlock the full potential of *C. asiatica* for treating numerous CNS disorders.

## 1. Introduction

Cognition can be defined as a set of mental processes that contribute to knowledge through experience, thought, and senses. A cognitive function comprises multiple fields, including concentration and attention, language, verbal memory, working memory, and visual memory [1]. Cognitive decline is one of the alarming health issues, which may cause illness, dementia, and death. Some aspects of age-related cognitive degeneration begin in healthy adults during 20–30 years [2]. Anti-inflammatory and antioxidant agents can be applied to restore mental and memory skills [3].

*Centella asiatica* (L.) Urban (*C. asiatica*) is a medicinal plant from India, Sri Lanka, China, Indonesia, and Malaysia [4]. It is called “pegaga” in Malaysia and is traditionally used to regenerate brain and nerve cells [5]*. C. asiatica* exhibited pharmacological profiles that correlated to the traditional use of *C. asiatica* in humans, which have neuroprotective properties, wound healing effects, and memory-enhancing properties [6]. The major phytochemicals of *C. asiatica* are pentacyclic triterpenes (madecassoside, asiaticoside, madecassic acid, asiatic acid) [7]. Additionally, it has been shown to promote brain growth and improve the memory and mental ability of intellectually disabled children [8]. Pharmacologically, *C. asiatica* demonstrated minimal toxicity in animal models' acute and subchronic toxicity tests [9]. *C. asiatica* extract has low absorption into the target due to the high molecular weight of active triterpenoids and is thus unable to cross the lipid membrane of the cells [10]. Therefore, *C. asiatica* is formulated into nanoemulsions to increase absorption and enhance its effectiveness. Nanocarrier phytodrugs have several advantages in natural product formulation. It has been reported that nanoemulsion formulation may help improve the pharmacokinetic and pharmacodynamic levels by enhancing absorption, bioavailability, and protection from toxicity [10]. Furthermore, nanotechnology has been applied to plant extracts because nanostructure systems could enhance the biological properties of plant extracts, decrease side effects, improve activity, enhance the consistent release of active constituents, and reduce the required dosage [7]. Nanoemulsion is a dispersion containing two immiscible colloidal dispersions liquid stabilized by surface-active agents, with a droplet diameter of 20 to 500 nm [11]. The advantages of nanoemulsion are having higher free energy, having long-term colloidal stability, and improving the bioavailability of the drugs [11]. The oil and water (O/W) nanoemulsions have a proclivity for solubilizing both hydrophobic and hydrophilic drugs in their structure by improving the delivery of drugs [12]. High-pressure homogenization may produce a fine stable emulsion at pressures in the range of 500–5000 psi [13].

The blood-brain barrier (BBB) proved to be an insurmountable barrier for several medications, such as central-nervous-system (CNS)-active drugs, antibiotics, and antineoplastic. It comprises a specialized vascular endothelium that interacts directly with astrocytes, neurons, and pericytes, restricting potential drug's penetrance of the central nervous system [14]. Furthermore, many drugs are ineffective in the CNS due to their low lipid solubility for BBB penetration [15]. Both parent compounds of *C. asiatica*, namely madecassoside and asiaticoside, have low lipid solubility that could restrict their ability to penetrate the intestinal membrane [16]. Thus, nanoemulsion is one of the approaches used as a drug delivery system to overcome problems in delivery systems such as poor target ability, low bioavailability, and improved penetrability across the intestinal membrane and BBB [17]. Virgin coconut oil (VCO) has been reported as a good oil phase due to its solvent capacity, good stability, and oxidation resistance, making it an ideal selection for the preparation of nanoemulsion [18]. Tween 80 (polysorbate 80, polyoxyethylene sorbitan monooleate) is a nonionic hydrophilic emulsifier commonly used in pharmaceutical and food products [19]. Furthermore, it has been used in conjunction with nanoparticles to improve the efficacy of doxorubicin, tacrine, loperamide, tubocurarine, and hexapeptide dalargin [19]. The presence of surfactants in the system, in turn, reduces the surface tension of the O/W phase to enable droplet formation, where their finite size makes it easy to penetrate through lipid membranes and deposit the bioactive compounds on the targeted site [20].

According to Samson et al. (2016), D-optimal mixture design has been practised in the pharmaceutical and food industries. The D-optimal mixture design has several advantages, including classifying the interaction to resolve the formulation process's shortcomings and reduce the number of experimental runs. Furthermore, it has been reported that the D-optimal mixture design was used to improve performance [21]. This study aims to optimize VCO-based nanoemulsion (oil, emulsifier, and water) using a D-optimal mixture design to obtain good size nanoemulsified particles. At the same time, interaction effects between variables and also factors that affect particle size were studied.

Nanoemulsion of ethanolic extract of *C. asiatica* (NanoSECA) was formulated using mathematical model formulation and physiochemically characterized for its stability, zeta potential, pH, morphology, and rheological behaviour. In addition, *in vitro* studies were also carried out on viability tests, acetylcholinesterase (AChE), inflammatory, and antioxidant activities. Furthermore, the ability of triterpenes (madecassoside, asiaticoside, madecassic acid, and asiatic acid), ethanolic extract of *C. asiatica* (SECA-K018), and NanoSECA to penetrate the BBB was evaluated using a Parallel Artificial Membrane Permeability Assay (PAMPA) kit. Results from this finding will lead to an alternative agent for the treatment of cognitive decline.

## 2. Materials and Methods

### 2.1. Materials

Virgin coconut oil was procured from Wellness Original Ingredients (Shah Alam, Malaysia), and polyoxyethylene sorbitan monooleate was purchased from Merck (Germany). A Milli-Q water purification system (EMD Millipore) was used to purify deionized water. The chemicals used were of analytical and food grade. Dimethyl sulfoxide (DMSO), 5,5′-dithiobis 2-nitrobenzoic acid, acetylthiocholine iodide, acetylcholinesterase (AChE), 3-(4,5-dimethylthiazol-2-yl)-2,5-diphenyl tetrazolium bromide, Hank's balanced salt solution, 2′,7′-dichlorofluorescin diacetate, dexamethasone, and physostigmine were purchased from Sigma-Aldrich (Germany). Madecassoside (purity ≥99%), asiaticoside (purity ≥98%), madecassic acid (purity ≥98%), and asiatic acid (purity ≥98%) were purchased from ChemFaces (Wuhan, China).

### 2.2. Preparation of Ethanolic Extract of *C. asiatica* (SECA-K018)

Raw materials of *C. asiatica* were collected from Ladang Pegaga, Kampung Kok Klang, Chuping, Perlis, Malaysia, and the herbarium specimen was deposited at the institution (UiTM; voucher specimen no.: CA-K018). Cleaned fresh leaves of *C. asiatica* (CA-K018) were crushed into pieces before drying in an oven at 40°C for three days. The dried plant materials were ground into a fine powder. The dried leaf powder (400 g) was macerated in 95% denatured ethanol (100 g/L) in a conical flask for three days at controlled room temperature. Then, the mixtures were filtered and the solvents were removed using a rotary evaporator under vacuum at 40°C. The samples were frozen at −80°C and then lyophilized in a freeze-dryer to remove residual water. The dried extracts were stored in glass bottles at 4°C before use. The extract was designated as the ethanolic extract of *C. asiatica* (SECA-K018), and the yield of extraction was 56.0%. Previously, the qualitative and quantitative analyses of SECA-K018 were performed based on triterpene compounds (madecassoside, asiaticoside, madecassic acid, and asiatic acid) using high-performance liquid chromatography (HPLC) [5].

### 2.3. Preparation of VCO-Based Nanoemulsion (VBN)

Limthin and Phromyothin (2019) performed a preliminary optimization of VCO-based nanoemulsion (VBN) of *C. asiatica*. In this study, we prepared a series of O/W nanoemulsions using a high-pressure homogenizer (Bee Technology) using the ingredients formulated from the experiment design matrix. Firstly, the combination of water, VCO, and Tween 80 was mixed using a magnetic stirrer at room temperature for 15 min to produce a homogeneous solution. The percentage (%) of Tween 80 was fixed at 10% throughout the optimization, as previously reported [22]. After that, the mixture was further homogenized using the high-pressure homogenizer at 4,000 rpm for 20 min.

### 2.4. Experimental Design

The authors investigated the effect of deionized water (A) and VCO (B) on the particle size of the nanoemulsion using a two-factor D-optimal design mixture.  Table 1 shows a list of independent variables and their coded stages.

A design matrix with 16 runs was generated, and the findings were statistically analyzed using Design-Expert version 7.0 by Stat-Ease Inc. (Minneapolis, USA). Following the D-optimal model design, each run's composition was randomized to reduce the impact of unexplained variability on the actual answer caused by extraneous factors. In addition, there were restrictions on composition *X*_*j*_ with a general form as in equation (1):

(1) $$\begin{array}{c} \sum Xj=1\text{ and }Lj\leq Xj\geq Uj.\; \end{array}$$

The lower (*L*_*j*_) and upper (*U*_*j*_) constraints were restricted to prevent analyzing the entire simplex region. Furthermore, the answer had to be based solely on the composition in the mixture rather than the total volume of the mixture [22]. We constructed the design matrix of the 16-point D-optimal experiment using Design-Expert 7.1.6 software (DX7; Stat-Ease, Inc.). In this study, the D-optimal design duplicated four design points to estimate the experimental error. The duplicated points (replicates) were Run #4 = #16 (vertices), Run 1 = #9 (vertices), Run #5 = #2 (vertices), and Run #12 = #13 (center edge). We randomized the order of the compositions of independent variables for each run to reduce unexplained variability in the actual answer.

### 2.5. Statistical Analysis

In this study, the authors used the D-optimal mixture design, which predicts the effects of different ingredient compositions against particle size, to find the best conditions for the independent variables. The best nanoemulsion compositions were selected based on the condition that resulted in the smallest particle size possible. Analysis of variance (ANOVA) and coefficient of determination (*R*^2^) were used to identify significant variations between the independent variables. A significant *p*-value (*p* < 0.05) is needed for an excellent final reduced model, and *R*^2^ greater than 0.9 is considered a model with a very high correlation [23].

### 2.6. Verification of Models

Various conditions with random formulations were considered to verify the model. The predicted and actual response values were used to measure the percentage of residual standard error. The final optimal composition of variables suggested was used to confirm the predicted optimum values of the model. Finally, the percentage prediction error (PPE) of VCO-based nanoemulsion is calculated using equation (2) to assess the adequacy of the final reduced model [20].

(2) $$\begin{array}{c} \text{PPE}\left(\%\right)=\left[\frac{\left(\text{Experimental}-\text{Predicted}\right)\text{Value}}{\text{Experimental Value}}\right]\times 100\%. \end{array}$$

### 2.7. Preparation of Nanoemulsion from SECA-K018

The best composition VCO-based nanoemulsion selected from optimization D-optimal mixture design was further subjected to speed optimization at various percentages of SECA-K018 (0.5, 1, 2, and 5%) [24]. The phytochemicals of SECA-K018 were analyzed by using HPLC analysis. It was found that SECA-K018 contained a high amount of madecassoside (179.64 mg/g), asiatic acid (132.26 mg/g), madecassic acid (112.82 mg/g), followed by asiaticoside (105.71 mg/g) [5]. A mixture of 2% (w/v) of SECA-K018 and the best composition of VCO-based nanoemulsion (designated as NanoSECA) was characterized and subjected to *in vitro* assessments.

### 2.8. Characterization of NanoSECA

#### 2.8.1. Particle Size, Zeta Potential, and Polydispersity Index (PDI) Analyses

The authors determined the particle size, zeta potential, and polydispersity index (PDI) of the NanoSECA using a Zetasizer Nano Series ZS90 and dynamic light scattering (DLS) (Malvern Instruments, Malvern, UK). The temperature controller (Julabo water bath) was set up at 25°C. The cumulant approach evaluates the autocorrelation function by fitting a single exponential to the correlation function to obtain particle size distribution. The particle size was calculated a day after the formulations were made to ensure that the system had reached equilibrium [25]. Scattering was measured in an optical quality 3 mL borosilicate cell at an angle of 90°C, where the samples were diluted in distilled water to reduce multiple scattering effects [22]. About 250 *µ*L of the emulsion was introduced to 50 mL of deionized water in a 100 mL beaker under gentle agitation [26]. The temperature was kept constant at 25°C [27].

#### 2.8.2. Morphology Study

We used transmission electron microscopy (TEM) to test the microscopic visualization of NanoSECA. The sample was dissolved in deionized water before being filtered via a 300-mesh screen. The sample was negatively stained using 1% (w/v) uranyl acetate. After that, the stained piece was allowed to dry at room temperature for 10 min. The grid was then observed from TEM after the excess liquid was dried with filter paper [15].

#### 2.8.3. pH Measurement

The pH of NanoSECA was determined by inserting the electrode (Mettler Toledo) directly into the sample solution at 24.0 ± 2.0°C [28].

#### 2.8.4. Rheological Measurement

The authors used a rheometer MCR 301 (Anton Paar Physica) fitted with a cone-and-plate test geometry (plate diameter 20 mm, cone angle 4) to analyze the viscoelastic properties of NanoSECA. All measurements were performed at 25°C [29]. The steady rheological activity of the sample was investigated at a regulated shear rate ranging from 0.1 to 100 s^−1^. Prior to measurements, the sample should stand for 10 min after loading to achieve an equilibrium state.

(3) $$\begin{array}{c} \eta =\;ky{`}^{n-1}, \end{array}$$

where *η* is the viscosity (Pa. s), *y*` is the shear rate (s^−1^), and *k* and *n* are the consistency index and flow behaviour index, respectively.

A rheometer MCR 301 (Anton Paar Physica) fitted with a cone and plate test geometry (plate diameter 20 mm, cone angle 4) was used to analyze the viscoelastic properties of NanoSECA. All measurements were performed at 25°C [29]. At a regulated shear rate ranging from 0.1 to 100 s^−1^, the steady rheological activity of the sample was investigated. Before measurements were taken, the sample should stand for 10 minutes after loading to achieve an equilibrium state.

(4) $$\begin{array}{c} \eta =\;ky{`}^{n-1}, \end{array}$$

where *η* is the viscosity (Pa. s), *y*` is the shear rate (*s* − 1), and *k* and *n* are the consistency index and flow behaviour index, respectively.

#### 2.8.5. Stability Study

The authors conducted experiments on the stability of NanoSECA under centrifugation and storage stability at different temperatures (4, 25, and 40°C for 0, 7, 14, and 30 days) [26]. The nanoemulsion was unstable if the visible appearance of creaming or phase separation occurred. Therefore, to test its stability under a centrifugation test, NanoSECA was kept in a centrifuge tube and subjected to centrifugation force (Eppendorf) at 4,000 rpm for 15 min. The optimized NanoSECA was then observed for any physical change and phase separation.

### 2.9. In vitro Assessments of NanoSECA

#### 2.9.1. Cell Culture

RAW 264.7 murine macrophages obtained from the American Type Culture Collection (ATCC no. CRL-2266 and no. TIB-71TM; Manassas, VA, USA) were maintained in Dulbecco's modified Eagle medium (DMEM) supplemented with 10% heat-inactivated fetal bovine serum (FBS) and 1% penicillin/streptomycin in a humidified incubator maintained at 37°C under 5% CO_2_/95% air.

#### 2.9.2. Determination of Cell Viability Assay

We investigated the cytotoxicity of NanoSECA on RAW 264.7 macrophages using an MTT (3-(4,5-dimethylthiazol-2-yl)-2,5-diphenyl-tetrazolium bromide) assay. A concentration of 30 × 10^3^ cells/mL (100 *µ*L/well) was seeded on 96-well plates. After 24 h, the cells were treated with various concentrations of NanoSECA and then incubated for 24 h at 37°C in an incubator with a humidified atmosphere of 5% CO_2_. MTT solution (5 mg/mL) was applied to each well at a rate of 20 *µ*L per well for 3–4 h at 37°C. In each well, formazan crystals were dissolved in DMSO. A microplate reader was used to measure absorbance at 540 nm to assess the strength of purple formazan [30].

#### 2.9.3. Determination of Anti-Acetylcholinesterase Activity

We used Ellman's method to assess the AChE-inhibitory function of NanoSECA, as previously reported [4]. Each sample (20 *µ*L of 5 mg/mL DMSO) was dispensed in triplicate into a 96-well microplate. First, 190 *µ*L of DTNB and 20 *µ*L of AChE were added and incubated for 15 min. Then, the substrate (ATCI) was added for another 15 min. The inhibitory activity of AChE was estimated at 412 nm at 30-s intervals. The formula used to measure the percentage inhibition of each sample and the positive control (physostigmine) is shown in equation (4):

(5) $$\begin{array}{c} \%\text{AChEI}:\left[\frac{\left(\text{Ac}-\text{As}\right)}{\left(\text{Ac}\right)}\right]\times 100\%, \end{array}$$

where Ac is the absorbance of control and As is the absorbance of the sample.

#### 2.9.4. Determination of Nitrite Production

RAW 264.7 macrophages were seeded 1 × 10^5^ cells/well in 12-well plates and incubated for 24 h. The cells were then incubated with various concentrations of NanoSECA (7.8125–1,000 *µ*g/mL) and LPS (4 *µ*g/mL) for 24 h. Dexamethasone was used as the positive control. As per a previously published method, Griess reagent was used to measure nitric oxide (NO) output in the culture medium [31].

#### 2.9.5. Determination of Measurement of Reactive Oxygen Species (ROS) Production

RAW 264.7 macrophages were seeded 30 × 10^3^ cells/mL in 96-well plates and incubated for 24 h. The cells were then incubated with various concentrations of NanoSECA (7.8125–1,000 *µ*g/mL) and LPS (4 *µ*g/mL) for 24 h. *α*-Tocopherol was used as the positive control. At the end of treatment, the medium was discarded and gently washed three times with Hank's balanced salt solution (HBSS). Next, the cells were stained with 20 *µ*M H_2_DCF-DA and left incubated for 45 min at 37°C in the dark. Then, the cells were gently washed three times with HBSS to remove excess dye solution. Finally, the fluorescence intensity was measured at an excitation wavelength of 485 nm and an emission wavelength of 520 nm after 200 *µ*L of HBSS was loaded into the wells [32].

### 2.10. Prediction of CNS Permeability

We used the PAMPA to assess the ability of compounds for brain penetration. The test compounds (10 mg each of madecassoside, asiaticoside, madecassic acid, and asiatic acid) were dissolved in DMSO. Meanwhile, SECA-K018 and NanoSECA were dissolved in DMSO at a concentration of 1,000 *µ*g/mL. 500 *µ*L of each sample, permeability control, and equilibrium standards were diluted with PBS. 300 *µ*L of PBS was added to wells in the acceptor plate. With the donor plate still in its tray, 5 *µ*L of 4% lecithin in dodecane was added directly to the well membranes of the donor plate. The donor plate was carefully placed into an acceptor plate well. Then, the plate was incubated at room temperature at 37°C for 18 h. After the incubation time, the donor plate was carefully extracted and the liquid was collected in the acceptor plate wells for analysis. This was referred to as an acceptor solution. The peak absorbance of samples was determined from the absorbance spectrum (200–500 nm) in 10-nm intervals. The blank control confirms that the peaks are due to test samples and not the DMSO in the solution. The peak absorbance values for high-permeability (HP), medium-permeability (MP), and low-permeability (LP) controls are 280 nm, 270 nm, and 270 nm, respectively [33]. The permeability rate (Pe) is determined using equation (5):

(6) $$\begin{array}{c} \text{Pe }=\;C\;\times \;-\ln\left(1-\frac{ODa}{ODe}\right)\frac{\text{cm}}{\text{s}}, \end{array}$$

where *ODa* is the absorbance of the acceptor solution, *ODe* is the absorbance of equilibrium standard, and *C* = 7.72 × 10^−6^ if the incubation runs for 18 h.

## 3. Results and Discussion

### 3.1. Optimization of Parameters for Nanoemulsion of *C. asiatica*

A preliminary study was performed to determine different deionized water and VCO percentages based on previous research on optimizing VCO-based nanoemulsion conducted by Limthin and Phromyothin (2019). This initial work was to identify suitable lowest and highest levels of each component in subsequent optimization experiment of D-optimal mixture design. VCO contains saturated medium-chain fatty acids, which are quickly metabolized by the body and contain antioxidants such as polyphenols, vitamins, and tocopherols [34]. The precipitation of active ingredients in water could be avoided by applying surfactants to the system. Polysorbate 80, known as Tween 80, is widely used for brain delivery as this surfactant promotes the adsorption of apolipoprotein *E* on the nanocarrier's surface [35].

The preparation of VCO-based nanoemulsion was monitored for particle size, as collectively, the parameters provided information on nanoemulsion's physical stability [36]. This is because nanoemulsion for targeted active ingredients delivery to the brain should have a droplet size between 20 and 200 nm or lower [35]. The PDI reflects the distribution and uniformity of the oil droplet. A small PDI of <0.25 indicates a concentrated and narrow particle size distribution and better stability [15]. For parenteral application, a PDI value up to 0.250 was acceptable [15]. PDI values of oral nanoemulsion formulation is less than 0.5, which indicates the uniformity of droplet size distribution and affirms their homogeneity as previously reported [37]. From the optimization analysis, the best formulation ratio was used for the preparation of nanoemulsion. The values of the variables used in the formulation are shown in Table 2. The upper and lower bounds on component proportion are needed for mixture design construction and analysis.

### 3.2. Model Fitting

This study performed two relevant variables: the deionized water (*A*) and VCO (B) of VCO-based nanoemulsion. According to the D-optimal design, the mean particle size of the nanoemulsion obtained experimentally is tabulated in Table 2. The predicted values agreed with the values obtained experimentally in almost all cases. In every case, the expected values matched the experimental values. Based on the experimental results, the particle size values were predicted. The final equation for the model that describes particle size is as in equation (6):

(7) $$\begin{array}{c} \text{Particle Size}=122.39-50.18\text{A}+196.81\text{B}\\ +13.88\text{AB}+97.11{\text{A}}^{2}+187.86{\text{B}}^{2}. \end{array}$$

Table 2 shows that the minimum particle size obtained was 129.76 nm (standard order: 2). Analysis of variance (ANOVA) was used to analyze the experimental data to determine the best-fitted model that can be used to determine the optimal composition for the preparation of NanoSECA. The statistical analysis for response (particle size), coefficient of determination (*R*^2^), adjusted coefficient of determination (R^2^_adj_), adequate precision, F-value, *p*-value, lack of fit, and coefficient estimate are listed in Table 3. Results revealed that the response was well fitted to a specific quadratic model.

**Figure 1:**
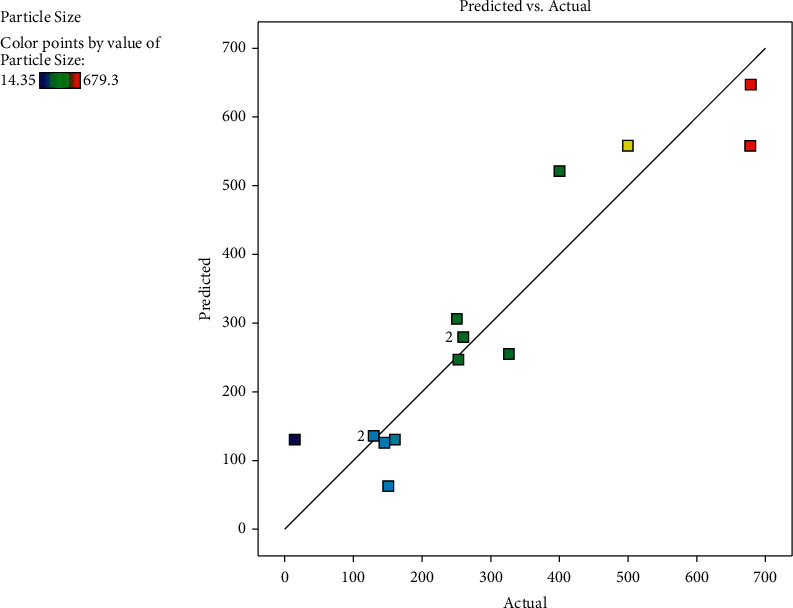
Predicted versus actual plot for particle size.

It is important to mention here that a *p*-value is deemed significant when it is <0.05. In this work, the comparable values of *R*^2^ and *R*^2^_adj_ for the models generated for particle size (*R*^2^ = 0.9244, *R*^2^_adj_ = 0.8866) were symbolic of good fitting between the regression model and experimental values (Nipornram et al., 2018). The plots substantiated in Figure 1 show good fitting between the predicted values versus actual values as obtained in this study. This was based on the normally distributed values and the absence of outliers in the straight-line plot. Thus, the model can adequately describe the correlation between the assessed variables and the response and predict the ratio for the smallest particle size of VCO-based nanoemulsion. Equally, the lack of fit (*p* > 0.05) for the particle size (*p*-value = 0.2365) was found insignificant in relevance to the pure error. Adequate precision (Adeq. Prec.: particle size = 13.473) (Table 3) indicated that the models could satisfactorily predict the best composition and navigate the design space to prepare nanoemulsion. High *R*^2^ values obtained from this study suggested that the quadratic model was highly efficient for fitting the data under the condition of experiment and the adjusted *R*^2^ values implied good agreement between the predicted and experimental values of the model for particle size. Furthermore, the *R*^2^ value of higher than 0.85 was adequate for prediction purposes. A greater value of adequate precision (more than 4) was preferred, indicating an adequate signal for the optimization process [38].

The model terms A, B, AB, A^2^, and B^2^ were significant for particle size as its *p*-value was <0.05. The positive and negative signs preceding all terms in equation (1) denote the antagonistic and synergistic effect, respectively, on the particle size.

**Figure 2:**
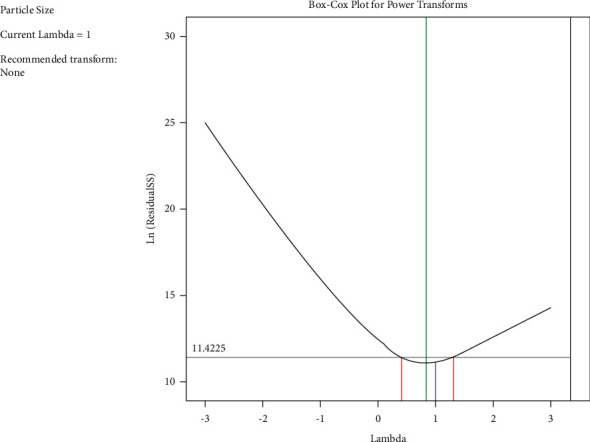
Box-Cox plot for particle size.

The Box-Cox plot proved the normality of the residuals, in which the lambda value describes the power raised by the transformation [39]. The ideal lambda value is at the lowest point of the Box-Cox plot, and the residuals were normally distributed (Figure 2). Since the predicted value for particle size (−0.4) was close to ideal values (1), it is concluded that transformation was unnecessary.

### 3.3. D-Optimal Analysis

**Figure 3:**
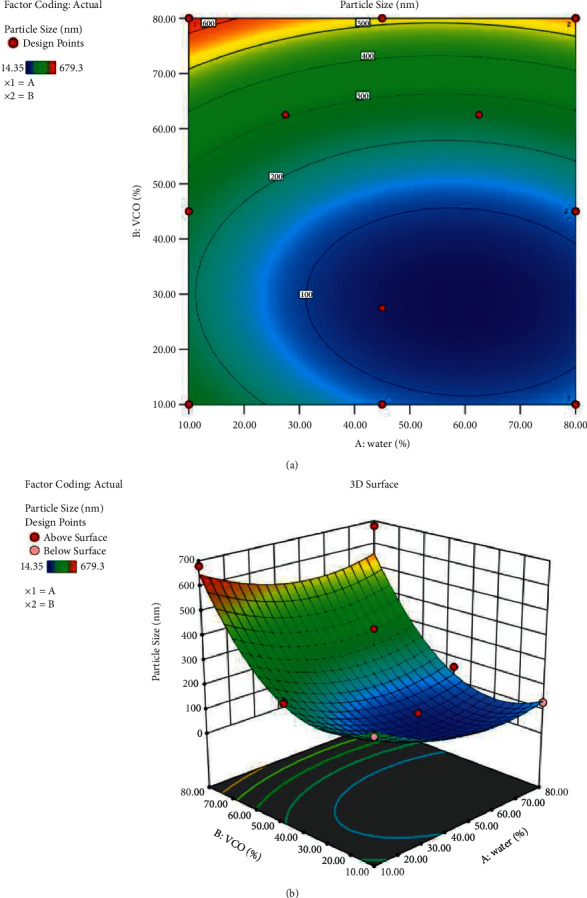
2D (a) and 3D (b) surface plots showed the interaction effect between 2 variables. A: water; B: VCO.

In this study, contour plots (2D) and three-dimensional (3D) plots were constructed to assess the effects of two variables on the particle size response of VCO-based nanoemulsion. As reported in model fitting, the interaction term AB for particle size response (*p*-value: 0.5794) showed lower F-values (F-value: 0.33), implying the low impact of lowering the particle size VCO-based nanoemulsion.  Figure 3(a) and 3(b) demonstrates the influence of water and VCO under constant composition of Tween 80, and the response surface plots present the significant effects of all two variables on the responses. Based on the 3D plot, the increment percentages of water until at a middle point reduced the particle size. However, an increase in the percentage of water and VCO increased the particle size (Figures 3(a) and 3(b)). Larger particle sizes occur when the viscosity in the system rises due to the efficiency of decreased particle dispersion [22]. This shows that the ratios of water and VCO must be carefully modulated to achieve the VCO-based nanoemulsion with a smaller particle size. The data obtained here were comparable with the findings of an earlier work by Limthin and Phromyothin (2019) when VCO was used in their formulation. Formulation of standard order: 2 was designated as VCO-based nanoemulsion: Formulation 2 (VBN: F2).

### 3.4. Predicted and Verification of Optimum Parameters

An optimum formulation condition can be defined as a desirable formulation obtained at specific conditions [39]. In this study, nanoemulsion for delivery to the blood-brain barrier should yield a minimum particle size (<200 nm) and PDI (<0.25). The best formulation VBN: F2 from Section 3.3 is further subjected to characterization analysis.

The optimum experimental values obtained from this study accorded well with the predicted particle size, giving reasonable predictive percentage errors (PPE) of both responses of <10% (Table 4), which indicated no significant difference between the actual and predicted values. This value also implied the validity and robustness of the attained mixture design model [40]. The optimum formulation of VBN: F2 was further optimized on the speed of the high-pressure homogenizer to reduce the particle size. The state of the surface of particle size and prediction of long-term stability can be measured by zeta potential. A highly stable formulation shows a value higher than ±30 mV [41]. Results revealed that the particle size (nm), zeta potential (ZP), and polydispersity index (PDI) for formulation VBN: F2: 10,000 decreased the particle size and narrowly distributed with a value of 93.42 nm and 0.225, respectively, which implied long-term formulation stability (Table 5). A high zeta-potential describes a formulation with a hydrophilic-lipophilic balance (HLB) value higher than 11. Hong et al. (2018) corroborated the benefits of using Tween 80 to formulate a nanoemulsion [41]. Hence, the VBN: F2: 10,000 rpm was used in the optimization percentages of SECA-K018.

### 3.5. Optimization of Percentage (%) of SECA-K018 in VBN: F2

Therefore, the applicability of the D-optimal MED to predict the percentage (%) of SECA-K018 showing the minimum droplet size and PDI was demonstrated (Table 6). VBN: F2: 10,000: (1%) of SECA-K018 showed the smallest particle size and the lowest PDI. However, it is not active in AChE-inhibitory activity as the IC_50_ value is more than 1000 *µ*g/mL compared with VBN: F2: 10,000: (2%) of SECA-K018. Thus, VBN: F2: 10,000: (2%) of SECA-K018 was selected in the subsequent experiments. Hence, 2% (w/v) of the SECA-K018 was adequate to deliver the active ingredients to the target. This model was reliable to optimize NanoSECA formulation based on stability and product acceptability. In addition, due to a large surface area and satisfactory dispersion offered by the nano-sized NanoSECA, rapid penetration of active ingredients targeted delivery to the blood-brain barrier was, therefore, the expected vital benefit. The result also revealed that NanoSECA was free from significant flocculation and agglomeration. Pertinently, a high energy barrier of particle size indicates uniform stability of the nanoemulsion, inferred from the negative zeta potential value [41]. The presence of the polyoxyethylene group also contributed to the negative symbol recorded for the zeta potential of NanoSECA and described the suitably acidic pH of the system [42]. The findings supported the adequacy of the D-optimal mixture design to optimize the composition of NanoSECA for the extended storage duration.

### 3.6. Characterization of NanoSECA

#### 3.6.1. Droplet Morphology by TEM Analysis

**Figure 4:**
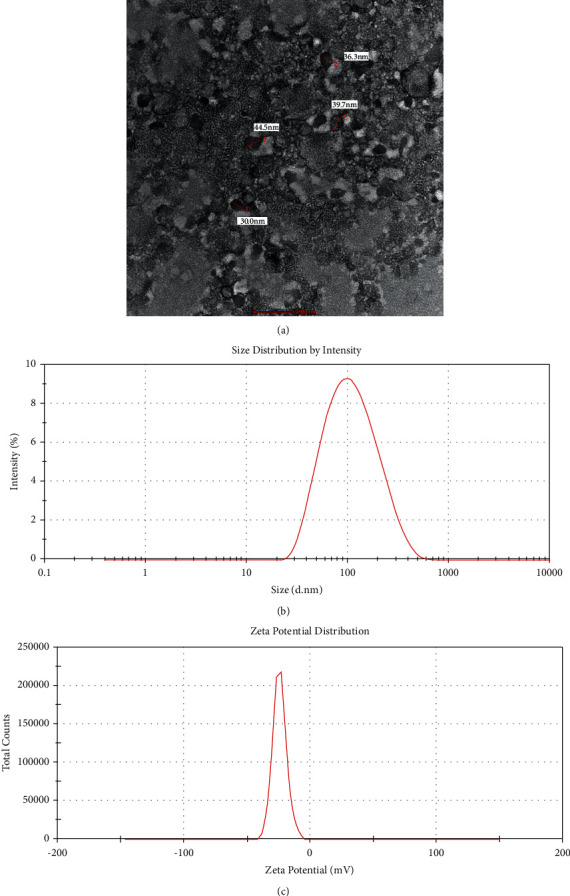
(a) TEM micrograph of NanoSECA, (b) particle-size distribution, and (c) zeta-potential distribution.

Morphology of the NanoSECA was investigated by air-dried negative staining transmission electron microscopy (TEM), and the micrograph showing the particle size morphology and distribution of NanoSECA at 100 nm magnification is depicted in Figure 4(a). Nano-sized spherical-shaped NanoSECA droplet was found to be between 30.0 nm to 44.5 nm. Overall, the results inferred satisfactory stability of the prepared NanoSECA, presumably from the tight-packed formed droplets [42]. Furthermore, the bold-black coloured droplets in the micrograph contained the NanoSECA active ingredients, as similarly observed by Kong et al. (2018) in the O/W nanoemulsion that they developed [43]. The range of particle size estimated by TEM (30.0 nm to 44.5 nm) is lesser compared to the dynamic light scattering (DLS) reading (127.83 nm) of the Zetasizer Nano ZSP instrument as liquid droplets in TEM tend to deteriorate under vacuum during the analysis of samples.

In contrast, the sample for DLS is analyzed in their hydrated forms in water and solvent. Droplet size determined by the DLS method is intensity-based, and it estimated the hydrodynamic diameter of droplets in NanoSECA and exhibited values more prominent than those measured by TEM. The sample tends to experience a certain degree of shrinkage in dried form [44]; hence, that explains the smaller droplet sizes of NanoSECA sample measured using TEM. Nonetheless, both types of analysis complement each other to determine the shapes and size of particle size within a system [45].

Figure 4(b) shows that the average particle size in the NanoSECA was 127.833 ± 8.280 nm, 70% of the particles were <100 nm and 40% < 400 nm in size, and the range of distribution was very narrow. We determined the PDI value, which measures the spread of the particle-size distribution, in which a small value indicates a narrow particle-size range.  Figure 4(c) shows that the average particle size in the NanoSECA was −24.9 ± 0.011 mV and remained in the −40 mV to −5 mV range.

#### 3.6.2. pH Analysis

The pH is essential for determining the stability of nanoemulsion due to the pH alteration occurrence of chemical reactions that can affect the quality of the product [46]. Therefore, oral nanoemulsions must be formulated within the recommended pH range of 4.9–6 to ensure the overall better quality of the formulation [47]. For this study, the pH of the NanoSECA was found to be at 6.0 ± 0.006, well within the acceptable pH value for oral nanoemulsion.

#### 3.6.3. Stability Tests

The stability of the NanoSECA in Tables 7 and 8 was assessed in terms of physical appearance, by which phase separation must be absent from reflecting the stability of nanoemulsion. In addition, in this study, the NanoSECA was tested under various storage conditions to predict the ability of the sample to withstand changes that may occur under market conditions [21].

Results revealed that NanoSECA remained stable after the centrifugation tests and unchanged visually. For the freeze-thaw test, NanoSECA was frozen at 4°C for 24 h and thawed at room temperature for another 24 h. The NanoSECA stayed stable despite drastic temperature changes, thereby corroborating the stability of nanoemulsion. Correspondingly, data for the physical stability of NanoSECA under different temperatures 4 ± 1, 25 ± 1, and 40 ± 1°C, for 30 days, were free from phase separation. Thus, an adequate amount of Tween 80 in the NanoSECA may have created a steric barrier between water and oil that prevented the destabilization of the system.

#### 3.6.4. Rheology

**Figure 5:**
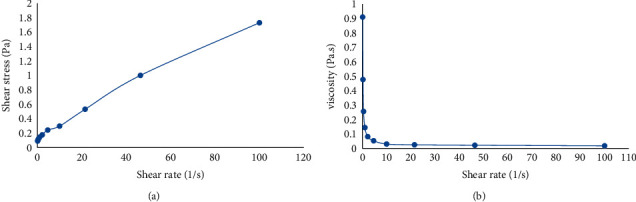
Flow curve: (a) shear stress and (b) viscosity versus the shear rate of NanoSECA.

The profiles for the shear stress-shear rate and viscosity-shear rate are presented in Figures 5(a) and 5(b), respectively. The NanoSECA showed shear-thinning and pseudoplastic as seen from the direct interaction in non-Newtonian behaviour [48] (Figure 5(a)). As the shear rate increased, the viscosity of the system decreased (Figure 5(b)). This behaviour demonstrated the breakage of the colloidal structure of the emulsion. However, the value of viscosity of NanoSECA might return to its initial value once the shear rate is reduced [48].

### 3.7. *In vitro* Assessments of NanoSECA

#### 3.7.1. Effect of NanoSECA on Cell Viability

**Figure 6:**
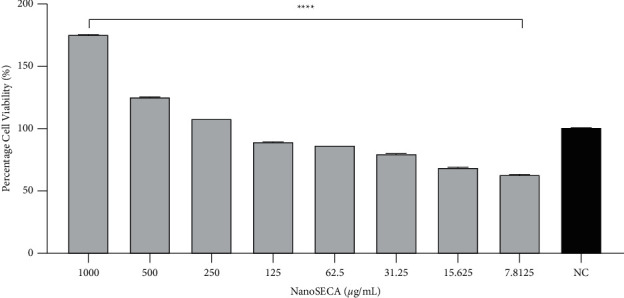
Percentage (%) cell viability of NanoSECA against SH-SY5Y cells after 24-h exposures using MTT assay at concentrations 7.8125–1000 *µ*g/mL; NC = negative control (untreated cells). Results were presented in triplicate as the mean ± SEM.  ^*∗*^ ^*∗*^ ^*∗*^ ^*∗*^*p* < 0.0001 compared with NC.

**Figure 7:**
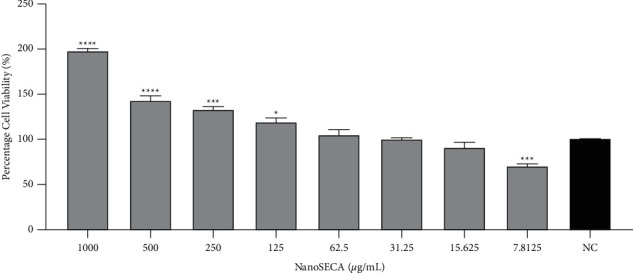
Percentage (%) cell viability of NanoSECA against RAW 264.7 cells after 24-h exposures using MTT assay at concentrations 7.8125–1000 *µ*g/mL; NC = negative control (untreated cells). Results were presented in triplicate as the mean ± SEM.  ^*∗*^*p* < 0.05, ^*∗*^ ^*∗*^ ^*∗*^*p* < 0.001, ^*∗*^ ^*∗*^ ^*∗*^ ^*∗*^*p* < 0.0001 compared with NC.

The MTT assay is a metabolic competence test that relies on mitochondrial performance to convert yellow MTT to a purple formazan derivative via mitochondrial respiration [49]. In this study, the cytotoxic effect of NanoSECA at different concentrations ranging from 7.8125 to 1000 *µ*g/mL was evaluated against SH-SY5Y and RAW 264.7 cell lines by using MTT assay (Figures 6 and 7). In addition, the effect of NanoSECA on SH-SY5Y and RAW 264.7 cells was evaluated after 24 h of cell exposure to the extract. Results revealed that the cell viability of NanoSECA increased in a dose-dependent manner and did not express any cytotoxic effect for both cells.

Based on results, NanoSECA did not exhibit toxicity against SH-SY5Y and RAW 264.7 cells. Since it did not exhibit toxicity against both cell lines at the concentration tested, these concentrations were selected for a subsequent experiment.

#### 3.7.2. Effect of NanoSECA on AChE-Inhibitory Activity

**Figure 8:**
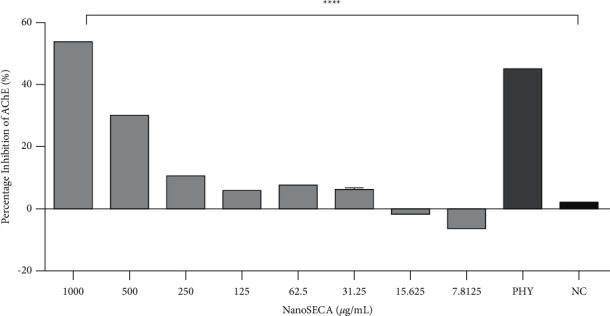
Effect of NanoSECA on AChE activity at the concentration 7.8125–1000 *µ*g/mL. NC = negative control (untreated cells); PHY = physostigmine (positive control). Results were presented in triplicate as the mean ± SEM.  ^*∗*^ ^*∗*^ ^*∗*^ ^*∗*^*p* < 0.0001 compared with NC.

The evaluation of the inhibitory effect of NanoSECA on AChE activity was carried out using Ellman's method as described by Zaidi et al., (2019) [50]. Acetylcholinesterase inhibitors (AChEIs), such as rivastigmine, galantamine, and physostigmine, have been considered symptomatic treatments for Alzheimer's disease [51]. In this work, physostigmine at 0.05 *µ*g/mL was used as the positive control throughout the assay. The neurotransmitter acetylcholine (ACh) is deficient in Alzheimer's patient's brains; inhibiting acetylcholinesterase (AChE), the primary enzyme that hydrolyses ACh, is one of the most effective treatments for neurodegenerative diseases [49]. Therefore, one of the targets to increase the level of neurotransmitters in the brain is by inhibiting AChE. Results demonstrated that NanoSECA efficiently inhibited the AChE enzyme in a dose-dependent manner (Figure 8). The IC_50_ value for NanoSECA was found at 974.9 ± 5.33 *µ*g/mL. This effect could be due to the presence of ketones in VCO responsible for oxidation of medium-chain triglycerides to increase the production of neurons [2].

Furthermore, Ovais et al. (2018) reported that asiatic acid and asiaticoside in *C. asiatica* help rejuvenate the neuronal cells, increasing intelligence, memory, and longevity. It is also supported by a literature survey that reported *C. asiatica* has comprehensive neuroprotection in which the mechanism of action by enzyme inhibition in neurodegenerative diseases [52].

#### 3.7.3. Effect of NanoSECA on Anti-Inflammatory Activity

Inflammation is a complex defensive mechanism that restores and neutralizes the normal function of cells and tissues. However, proinflammatory induction and stress conditions lead to chronic diseases [53]. Macrophages are responsible for secreting inflammatory cytokines after activation and mediating inflammation [54]. Lipopolysaccharide (LPS) is a part of Gram-negative bacteria's outer wall that activates macrophages and leads to the release of specific proinflammatory cytokines such as promoting tissue damage and chronic diseases [54].

**Figure 9:**
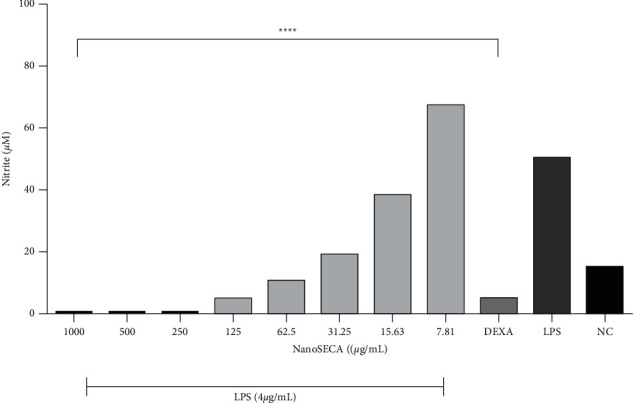
Effect of NanoSECA on nitrite production. NC: negative control (untreated cells); Dexa: dexamethasone (positive control); LPS: lipopolysaccharide. Results were presented in triplicate as the mean ± SEM.  ^*∗*^ ^*∗*^ ^*∗*^ ^*∗*^*p* < 0.0001 compared with NC.

RAW 264.7 cells were used to assess the anti-inflammatory effect of NanoSECA throughout the assay. NO production was determined on NanoSECA-treated RAW 264.7 cells exposed to LPS. Interestingly, results showed that NO production in the LPS-stimulated cells at 4 *µ*g/mL was suppressed by NanoSECA (15.64–1000 *µ*g/mL) in a dose-dependent manner (Figure 9). Dexamethasone at 5 *µ*M was used as a positive control. It has been reported that asiaticoside found in *C. asiatica* had anti-inflammatory properties in LPS-stimulated RAW 264.7 cells [55]. NanoSECA exhibited nitrite's most significant maximal 50% inhibitory effect with a value of 3.159 ± 1.038 *µ*g/mL.

#### 3.7.4. Effect of NanoSECA on Antioxidant Activity

**Figure 10:**
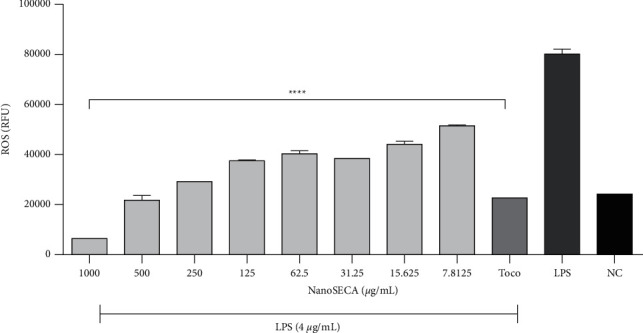
Effect of NanoSECA on ROS production. NC: negative control, Toco: *α*-tocopherol (positive control); LPS: lipopolysaccharide. Results were presented in triplicate as the mean ± SEM.  ^*∗*^ ^*∗*^ ^*∗*^ ^*∗*^*p* < 0.0001 compared with NC.

*C. asiatica* has been reported to improve cognitive impairment related to ageing and other neurodegenerative disorders such as Alzheimer and dementia [56]. Results revealed that NanoSECA significantly attenuated ROS production in a dose-dependent manner (Figure 10). The suppression of oxidative stress would be beneficial against cognitive decline and memory loss [57].

Previous findings suggested that *C. asiatica* has cognition-enhancing properties to protect against oxidative stress as it could reduce mitochondrial damage and increase neurite elongation and axonal regeneration [58]. Mitochondria generate reactive oxygen species (ROS) due to mitochondrial electron flow in the respiratory chain [59]. However, excessive ROS could trigger mitochondrial damage. Based on the above discussion, NanoSECA could potentially reduce the intracellular ROS production induced by LPS. *C. asiatica* has been widely reported to have neuroprotective effects and neuronal antioxidants *in vitro* and *in vivo* [60]. It has been reported that triterpenoid acids such as madecassic acid and asiatic acid found in *C. asiatica* decreased oxidative stress [61]. NanoSECA was found to exhibit a maximal 50% inhibitory effect against ROS with a value of 586.7 ± 2.768 *µ*g/mL.

### 3.8. Prediction of CNS Permeability

The PAMPA measures passive permeability using artificial membranes [16]. *C. asiatica* is believed to have beneficial biological activities such as antioxidative properties to attenuate oxidative stress, a high anti-inflammatory action, neurotoxicity inhibition effect, antidepressive and antianxiety properties, and AChE-inhibitory potential. Furthermore, its extensive multifunctional properties can target various disease mechanisms, including neurodegenerative diseases. As a result, an effective therapeutic agent based on *C. asiatica* active ingredients should be developed to penetrate the BBB.

Most drugs used to treat neurodegenerative diseases are lipophilic molecules with molecular weights greater than 400 Da, making them unable to pass through the BBB [62]. The BBB comprises a monolayer of endothelial cells bound by complex tight junctions, and neurons, astrocytes, and pericytes regulate its function. The complexity of BBB restricts the entry of active ingredients inside the brain [14]. Recently, nanotechnology-based drug delivery has been applied to overcome these obstacles and enhance drug delivery effectiveness across BBB [17]. The apparent oral bioavailability of madecassoside and asiaticoside was less than 1% due to larger molecular sizes and the presence of sugar moiety in their structures [16]. A recent study showed that madecassoside, asiaticoside, and asiatic acid are highly BBB permeable towards *in vitro* BBB models from primary porcine brain endothelial cells (PBECs) [63]. However, there are no reported findings on *C. asiatica* extract and nanoemulsion formulation. The results obtained from the assay corresponded with the prediction of CNS permeability, as shown in Table 9.

This study results revealed that *C. asiatica* bioactive compounds, namely, asiatic acid, madecassic acid, asiaticoside, and madecassoside, are capable of crossing the BBB. This study used two known marketed drugs: CNS− (enoxacin) and CNS+ (verapamil) as reference drugs. This is mainly based on their ability to cross the BBB. Based on the findings, NanoSECA showed high permeability (CNS+) with higher BBB permeation values (Pe: 15.19 ± 0.3 × 10^−6^ cm s^−1^) than four triterpenes and positive control (enoxacin). It would be a better approach for future studies to conduct an *in vitro* model and high-precision analytical instrument to validate the permeability studies of NanoSECA.

## 4. Conclusion

This study demonstrated that the D-optimal mixture design is an effective tool for optimizing VCO-based nanoemulsion for the formulation of NanoSECA. The variance analysis showed the model's fitness with a low *p-*value (<0.0001) and a nonsignificant lack of fit. The model also showed a high coefficient of determination of *R*^2^ = 0.9244. The optimal composition of VCO-based nanoemulsion was established as 80% of water (w/v) and 10% of VCO (w/v) that produced a lower particle size. In addition, NanoSECA is able to cross the BBB and improve the permeability rate towards the artificial membrane system. Furthermore, the protective effect of NanoSECA against LPS-induced oxidative stress and inflammation in the RAW 264.7 cells provides valuable data to support the therapeutic development of NanoSECA.

## Figures and Tables

**Table 1:** Constraints of independent variables.

Independent variables	Lower limit (%)	Upper limit (%)
Deionized water (A)	10.0	80.0
VCO (B)	10.0	80.0

**Table 2:** Experimental data of actual and predicted values of particle size of VCO-based nanoemulsion.

Standard order	Run order	Independent variables		Particle size (nm)	
		Deionized water (%)	VCO (%)	Actual^1^	Predicted
1	4	10.00	10.00	259.93 ± 0.577	274.61
2	1	80.00	10.00	129.76 ± 1.762	146.49
3	5	10.00	80.00	679.30 ± 1.315	640.48
4	12	80.00	80.00	500.23 ± 0.252	567.87
5	11	45.00	80.00	400.45 ± 2.261	507.06
6	10	80.00	45.00	160.20 ± 6.102	169.31
7	7	45.00	27.50	150.36 ± 10.344	70.950
8	14	10.00	45.00	253.20 ± 3.710	269.68
9	3	27.50	62.50	250.80 ± 2.301	313.66
10	15	62.50	62.50	326.60 ± 5.803	270.41
11	6	45.00	10.00	145.65 ± 2.301	113.44
12	16	10.00	10.00	259.93 ± 5.803	274.61
13	9	80.00	10.00	129.76 ± 0.987	146.49
14	2	10.00	80.00	678.10 ± 2.984	640.48
15	13	80.00	80.00	500.23 ± 3.176	567.87
16	8	80.00	45.00	140.35 ± 2.506	169.31

^1^Data obtained are means of triplicates.

**Table 3:** ANOVA and regression of particle size.

Source	Coefficient estimate	F-value	*p*-value	Significance
Model	—	24.46	<0.0001	Significant
A	−50.18	5.93	0.0351	
B	196.81	87.11	<0.0001	
AB	13.88	0.33	0.5794	
A^2^	97.11	5.53	0.0406	
B^2^	187.86	21.19	0.0011	
Lack of fit		1.98	0.2365	Not significant
*R* ^2^			0.9244	
R^2^_adj_			0.8866	
Adeq. Precision			13.473	

**Table 4:** Predicted percentage errors (PPE) for the obtained optimum composition for particle size of VBN: F2.

VBN: F2	A	B	C	Y_1_		
				Act.	Pred.	PPE (%)
	80	10	10	129.76	146.49	−12.02

^a^A: percentage of water), B: percentage of VCO, C: percentage of Tween 80, Y1: particle size (nm).

**Table 5:** Optimization data on speed of VBN: F2.

No.	Formulations (rpm)	Particle size (nm)	Zeta potential (mV) (ZP)	Polydispersity index (PDI)
1	VBN: F2: 6,000	135.267 ± 3.400	−19.400 ± 1.706	0.393 ± 0.008
2	VBN: F2: 8,000	101.570 ± 0.416	−28.633 ± 3.515	0.263 ± 0.005
3	VBN: F2: 10,000	93.420 ± 0.467	−30.767 ± 0.351	0.225 ± 0.008

**Table 6:** Optimization data on percentage (%) of SECA-K018 in VBN: F2.

No	Formulations (% of SECA-K018)	Particle size (nm)	Zeta potential (ZP)	Polydispersity index (PDI)	AChE-inhibitory activity (IC_50_) *µ*g/mL
1	VBN: F2: 10,000: (0.5%)	147.50 ± 3.236	−19.567 ± 0.015	0.386 ± 5.123	>1000
2	VBN: F2: 10,000: (1%)	92.397 ± 4.080	−27.833 ± 0.874	0.259 ± 0.070	>1000
3	VBN: F2: 10,000: (2%)	127.833 ± 8.280	−24.9 ± 0.011	0.493 ± 4.681	974.9 ± 5.33
4	VBN: F2: 10,000: (5%)	192.767 ± 13.249	−1.375 ± 1.005	0.809 ± 0.057	>1000

**Table 7:** Summary on centrifugation and freeze-thaw stability of NanoSECA.

Stability test	Centrifugation	Freeze-thaw (cycles)							
		1		2		3		4	
Temp (˚C)	-	4	25	4	25	4	25	4	25
Stability	√	√	√	√	√	√	√	√	√

^a^√: stable, ×: unstable.

**Table 8:** Summary on stability at different temperatures of NanoSECA.

Day (s)	0			7			14			30		
Temp (˚C)	4	25	40	4	25	40	4	25	40	4	25	40
Stability	√	√	√	√	√	√	√	√	√	√	√	√

^a^√: stable, ×: unstable.

**Table 9:** Permeability of commercial drugs in the PAMPA-BBB assay (used in experiment validation), NanoSECA, and their predictive CNS penetration.

Compound/Extract	PAMPA-BBB (Pe; 10^−6^ cm s^−1^)		Prediction^4^
	Reported permeability^2^	Experimental permeability^3^	
Enoxacin	0.9	0.2 ± 0.1	CNS−
Verapamil	16.0	16.0 ± 5.0	CNS+
SECA-K018	—	4.5 ± 0.8	CNS+
NanoSECA	—	15.19 ± 0.3	CNS+
Madecassoside	—	0.26 ± 0.7	CNS−
Asiaticoside	—	0.26 ± 1.0	CNS−
Madecassic acid	—	0.27 ± 0.3	CNS−
Asiatic acid	—	0.3 ± 0.3	CNS−

^1^PBS: DMSO (95 : 5) was used as a solvent. ^2^Reference (Chlebek et al., 2019). ^3^Data are the mean ± SEM of independent experiment. ^4^CNS+/−, BBB permeation uncertain; Pe, 10–6 cm s^−1^ from 4.0 to 2.0; CNS+, high BBB permeation predicted; Pe, 10–6 cm s^−1^ > 4.0; CNS−, low BBB permeation predicted; Pe, 10–6 cm s^−1^ < 2.0 (Di et al., 2003).

## Data Availability

No additional data are available.
